# Highlights from the 13th African Continental Meeting of the International Society of Paediatric Oncology (SIOP), 6–9 March 2019, Cairo, Egypt

**DOI:** 10.3332/ecancer.2019.932

**Published:** 2019-05-28

**Authors:** Elhamy Rifky Khalek, Glenn M Afungchwi, Mohamed El Beltagy, Ndagire Mariam, Hoda Zaki, Trijn Israels, Elizabeth Molyneux, Scott C Howard, Catherine Patte, Judy Schoeman, Elena Ladas, Mohamed S Zaghloul, Yasser S ElDeen, Soha Ahmed, Sherif Kamal, Eric Bouffet, Kathy Pritchard-Jones, Laila Hessissen

**Affiliations:** 1 Professor of Paediatric Oncology, Faculty of Medicine, Zagazig University, Children’s Cancer Hospital Egypt 57357, Cairo 11617, Egypt; 2 Manager, Cameroon Baptist Convention Health Services Childhood Cancer Programme, Cameroon University of Stellenbosch, Stellenbosch 7602, South Africa; 3 Professor of neurosurgery, Faculty of Medicine, Cairo University, Kasr El Aini, Cairo, and Head of Neurosurgery, Children’s Cancer Hospital Egypt 57357, Cairo 11617, Egypt; 4 Nursing officer, Uganda Cancer Institute, Kampala, PO Box 3935 Uganda; 5 Dean, Faculty of Nursing, Helwan University, Modern University for Technology and Information (MTI), Cairo, Egypt; 6 Princess Màxima Center for Pediatric Oncology, 3584 CS Utrecht, The Netherlands; 7 Paediatric Department, College of Medicine/Queen Elizabeth Central Hospital, Blantyre, Malawi; 8 The University of Tennessee Health Science Center, Memphis, TN 38163 USA; 9 CLCC G Groupe Franco-Africain d’Oncologie Pédiatrique (GFAOP), Institut Gustave Roussy, France and GFAOP, 94800 Villejuif, France; 10 Chief dietician, Department of Paediatrics and Child Health, Stellenbosch University, Cape Town 7530, South Africa; 11 Associate Professor for Global Integrative Medicine, Director, Integrative Therapies Program, Columbia University Medical Center, USA; 12 Professor of Radiation Oncology, National Cancer Institute; Chair, Radiation Oncology Department, Children’s Cancer Hospital Egypt 57357, Cairo, Egypt; 13 Professor of Pediatric Surgery, Alexandria University, Alexandria 21568, Egypt; 14 Chairman, Clinical Oncology Department, Aswan University and Consultant, Children’s Cancer Hospital Egypt 57357, Cairo, Egypt; 15 Director, Department of Pharmaceutical Services, Children’s Cancer Hospital Egypt 57357, Cairo, Egypt; 16 President, International Society of Paediatric Oncology (SIOP), Professor of Neuro-oncology, Sick Children’s Hospital, Toronto ON M5G 1X8, Canada; 17 SIOP President-elect, Professor of Paediatric Oncology, UCL Great Ormond Street Institute of Child Health, University College London, London WC1E 6BT, UK; 18 SIOP Africa Continental President, Professor of Paediatrics, Pediatric Haematology and Oncology Center, University Mohamed V Rabat, Rabat BP.8007.UN, Morocco

**Keywords:** childhood cancer, clinical pharmacy, nutritional assessment, paediatric cancer nursing, paediatric neurosurgery

## Abstract

The 13th African continental meeting of the international society of paediatric oncology, held on 6–9 March 2019 in Cairo, was organised in collaboration with the Children Cancer Hospital (57357) in Egypt and the global parents’ organisation (Childhood Cancer International) and supported by a large international faculty. With 629 delegates from 37 countries (24 African), this was the largest forum of healthcare professionals focused on children and young people with cancer in Africa to showcase advances and discuss further improvements. Three targeted workshops, on nursing care, pharmacy and nutrition, attracted large numbers and catalysed new collaborative initiatives in supportive care studies, extended roles for pharmacists in quality control and care delivery and addressed malnutrition concurrently with cancer treatment. The Collaborative Wilms Tumour Africa Project, open in seven sub-Saharan countries, and the trials in Burkitt’s lymphoma reported encouraging outcomes with further initiatives in supportive care (the supportive care for children with cancer in Africa project). While acknowledging deficits in radiotherapy provision, available in only 23 of 52 African countries, centres with facilities reported their technical advances that benefit patients. Of great importance for children with brain tumours, who are underdiagnosed in Africa, was the first announcement of African paediatric neuro-oncology society, whose 63 current members aim to tackle the shortage of neurosurgeons through training fellowships, workshops and a dedicated conference. The congress provided the opportunity to discuss how African countries will work with the WHO global initiative aiming to improve childhood cancer survival to 60% in all countries by 2030. This conference report is dedicated to the three Kenyan delegates who died tragically on the Ethiopian Airlines flight ET302 on their way home, full of new ideas and pride in what they had achieved so far. All those who heard their presentations are determined to continue their excellent work to improve cancer care for children in Africa.

## Introduction

Successful treatment of childhood cancers in Africa is of increasing importance due to the high proportion of children and adolescents in the African population and the continuous decrease in death rates from other causes [1]. This 13th biannual continental meeting of the international society of paediatric oncology (SIOP), in conjunction with African representatives from parents’ organisations [Childhood Cancer International (CCI)] and the Children’s Cancer Hospital (57357) in Egypt (CCHE), Cairo, aimed to showcase progress in this field across multiple disciplines and received 340 abstracts from more than 33 countries, many describing very positive progress through collaborative prospective clinical research [2].

Delegates were welcomed to the congress by Prof Elhamy Rifky A.Khalek (President of the Conference and the host of the event), Prof Laila Hesssissen, President of SIOP Africa and the members of the Local Organising Committee. Prof Rifky welcomed the participants and gave a brief summary of the congress and the schedule. The congress was attended by a total of 629 delegates from 37 countries (24 African), including 156 paediatric oncologists, 53 paediatricians, 32 radiotherapists, 27 surgeons, 92 nurses, 59 pharmacists, 14 diagnostic services (including nine pathologists), 102 nutritionists and 38 parents. Scholarships were available for 156 medical and nursing delegates, for which support from Sanofi Espoir Foundation, CCI, CCHE (57357), Egyptian National Cancer Institute, Ministry of Public Health, Pfizer Pharmaceuticals, New Bridge and Abbott Nutrition is acknowledged.

Professor Sherif Aboul Naga (CCHE, Cairo, Egypt) described in the opening ceremony how the CCHE 57357 (CCHE, widely known as Hospital 57357) was created, inspired by the model of the St. Jude Research Hospital in Memphis. The people of Egypt and friends from all over the world and most particularly in the Arab World generously contributed, and it was built completely by donations. The hospital’s mission is to provide the best comprehensive family-centred quality care and a chance for cure to all children with cancer seeking its services, free of charge and without discrimination. It opened in 2007 with 179 beds and, by 2018, had grown to 320 beds and has over 15,000 patients under active treatment. It has all the ‘state-of-the art’ clinical facilities (including two linear accelerators with plans for proton beam therapy) and comprehensive support services, in-house schooling and child life and play. Since its inception, the CCHE leadership realised that carrying out research in medical and non-medical areas was a prerequisite to progress in achieving cures and a better future for children with cancer. Hence, the adoption of an advanced health informatics system, which enabled it to be a paperless hospital, with the complete digitalisation of operational aspects and acquisition of a strong database. They made a significant and transformational investment in clinical pharmacy staff and processes. He emphasised the importance of investing in people, with all staff given time for and expected to contribute to research and education. Leadership training and embedding key performance indicators at all levels, with regular targeted feedback to departments and teams, have enabled the organisation to make remarkable progress in improving survival rates to an estimated 73% average overall survival rate for those treated today.

One of the biggest barriers for delegates who wished to attend was obtaining a visa in a timely fashion. Of the 33 participants affected, some of whose work had been selected for prize consideration, only 22 were able to obtain a visa on time to attend. Visas were only issued after personal interventions by the local organising committee, adding considerably to the administrative burden of organising a clinical conference in Africa. This issue needs to be considered by both future delegates and conference organisers, to ensure timely sharing of learning to benefit children with cancer and the healthcare professionals who care for them.

**Figure 1.**
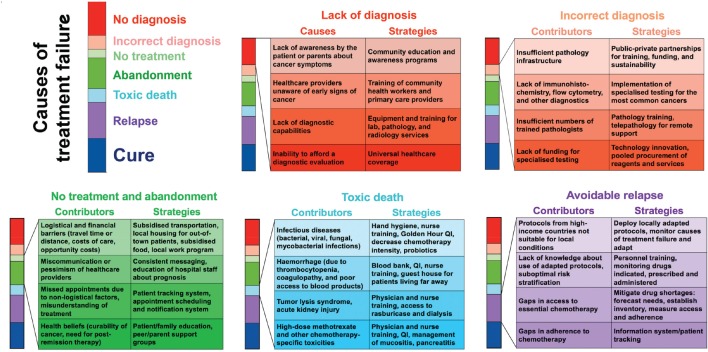
Cause-specific interventions to reduce treatment failure for children with cancer in low- and middle-income countries. (Used with the permission of Scott C Howard, MD, MSc.)

The programme covered almost all aspects of childhood cancer care, from improving diagnosis to delivering successful treatment adapted to the available resources. The importance of working collaboratively and involving parents to define needs was emphasised to demonstrate that, even in the most resource-challenged settings, progress in survival rates and quality of care can be achieved through targeted interventions (Figure 1). Three dedicated workshops in the key areas of nursing, pharmacy and nutrition, described in detail in the following section, were very well attended and focused on the specific challenges faced by African children with cancer and the paediatric services who care for them. The congress highlighted the impressive progress made through prospective clinical trials and studies and how this research effort has reaped wider benefits for paediatric care and built durable collaborative research networks. Further information is available from [2, 3].

## Nursing workshop

The nursing programme at SIOP Africa Cairo 2019 comprised two full days of workshop, keynote lectures, free-paper sessions and discussions of collaboration, attended by nurses from seven countries. A workshop on nursing research was delivered by Dr Faith Gibson, laureate of the SIOP Nurse lifetime achievement award 2018. Dr Gibson taught the nursing group how to identify useful research topics, various types of quantitative and qualitative research methodologies suitable for answering multiple research questions and research steps from planning to the dissemination of findings. Nurses expressed several areas of research priorities, a list of which was collated for further exploration within the group.

Three keynote lectures were delivered. Prof Nagwa Elkhateb from Egypt emphasised the importance of pain assessment using culturally and age-appropriate tools, followed by the meticulous pharmacological or non-pharmacological intervention. Sr Rachel Hollis (Leeds Hospitals NHS Trust, UK) gave a keynote lecture on the SIOP Paediatric Oncology in Developing Countries (PODC) nursing baseline standards for low- and middle-income countries (LMICs) and advocacy. These six standards for quality nursing care include staffing based on patient acuity; formal orientation programmes; continuous education; recognition of nurses as integral members of the multidisciplinary teams; resources for safe care; and research for evidence-based nursing practise [4]. A recent survey showed that the disparities in the attainment of the baseline standards with LMICs were largely disadvantaged [5]. An advocacy toolkit for these standards is available on the SIOP website [6]. Prof Zeinab Lotfy (Modern University for Technology and Information, Cairo, Egypt) of Egypt talked about the essence of communication skills in nursing education, highlighting the need for the consideration of local cultural realities in important aspects of nursing care such as breaking bad news and educating children and families on their treatment.

There were 11 free papers, three of which were recognised for their quality and relevance. Joan Nakabiri (Uganda Cancer Institute, Kampala, Uganda) from Uganda presented on how a continuous nurse’s education programme has increased the knowledge and confidence of paediatric oncology nurses at the Uganda Cancer Institute. Hany Eskander from CCHE was recognised for an assessment of intensive care nurses’ knowledge and practises regarding utilisation of infection control standards which showed a positive correlation between knowledge and practise of infection control [7]. He recommended continuous education on the latest evidence-based infection control practises. Finally, Vera Njamnshi (Cameroon Baptist Convention Health Services, Cameroon) from Cameroon was recognised for presenting on the contribution of the nurses’ role in planning, patient follow-up, informed consent and data collection for assessing the fertility of long-term female Burkitt lymphoma survivors.

Collaboration was one of the central themes of the nursing programme. In order to facilitate communication and sharing of knowledge and initiative in-between conferences, the nurses decided to create SIOP Africa nursing WhatsApp and Facebook groups. An evaluation form completed by most participants showed that they were satisfied with its various components. A few suggestions for future meetings were: to include more content related to palliative care and psychosocial support, to arrange sitting in a U-shape for better interaction and to allocate more time for group work.

## Clinical pharmacy workshop

For the first time in Africa, a one-day workshop was held to bring together all those working in clinical pharmacy services and those prescribing chemotherapy for children with cancer. Sessions were interactive with networking resulting in several future cooperative projects—in particular, those that empowered pharmacists in Africa to enhance their role to improve safety and efficacy of treatment for children with cancer and to use resources more efficiently. Dr SA Naga, the founder of clinical pharmacy in Egypt, opened with the history of the clinical pharmacy concept, the recognition of its value and examples of practical implementation in Egypt. Klaus Meier (HKK (Heidekreis-Klinikum GmbH Krankenhaus), Soltau, Germany), current President of the European Society of Oncology Pharmacy (ESOP), presented the ESOP’s plan to develop oncology pharmacy practise over a period towards 2025, including the launch of a certification programme for Oncology Pharmacists comprising 100 hours of training including webinars and face-to-face international and national educational activities. He discussed how the current ESOP programme includes oral chemotherapy, QUAPOS (oncology pharmacy practise standards), the contamination project, safe handling and clean working, the essential requirement for oncology practise, the EUSOP certification programme and, finally, the ECOP conference in Malta. Both speakers urged oncology pharmacists in Africa to unite and work together to implement the best evidence-based pharmacy practise.

The surgical session was well attended by different generations of different sub-specialties including paediatric surgeons, paediatric oncologists and paediatric radiotherapists from different institutes from all over Egypt as well as different African countries. The session was also enriched by fruitful discussions following each presentation. One of the main recommendations during these discussions was to encourage multicentric studies and surveys suggested by physicians and researchers interested in cancer children with all of its different specialties in Egypt. It was proposed that a future conference should ensure greater attendance by international paediatric oncology surgical faculty from the International Society of Paediatric Surgical Oncology (IPSO).

## Nutrition workshop

Malnutrition is widespread among children living in Africa with approximately 46% of children diagnosed with cancer also being diagnosed with malnutrition [8]. Managing malnutrition can be challenging for paediatric cancer units (PCUs) with limited resources [9]; however, the clinical implications of not remediating malnutrition leads to reduced survival and increased treatment-related toxicities [10]. On the final day of the conference, a nutrition workshop was convened, which included dieticians, nurses, physicians, parent groups and nongovernmental organisations (NGOs). Dr Elena Ladas (Columbia University, USA) and Dr Ronald Barr (McMaster University, Canada) opened the workshop with presentations on the impact of nutritional status on survival and outcome and the importance of performing sequential nutritional assessments throughout treatment. An important highlight was the ease and use of mid-upper circumference (MUAC) to determine nutritional status. Regional data on nutritional status, and barriers to care, were provided by clinicians in Ethiopia (Dr Daniel Hailu), South Africa (Judy Schoeman), Malawi (Dr Trijn Israels) and Egypt (Dr Sahar Khairy). Striking figures on the rates of malnutrition among children with cancer were presented; for example, in Malawi, incidence reaches 95% when MUAC or triceps skinfold thickness is utilised for nutritional assessment.

Limited access to nutritional products has been reported among PCU in Africa [9]. Ms Bella Beryl Jamona (Hope for Cancer Kids, Kenya) discussed the challenges clinicians face in providing optimal care to Kenyan children. Prof Mariana Kruger (Tyerberg Children’s Hospital, Stellenbosch, South Africa) (South Africa) and Dr Lillian Gesami-Steytler (Windhoek, Namibia) presented on limited access to enteral products and challenges faced when implementing ready-to-use therapeutic formulas. A persistent barrier was the poor availability of these products in PCUs and the lack of trained personnel able to manage children with cancer when they also have severe acute malnutrition. Several case studies illustrated varied approaches to the delivery of nutritional care in a limited resource setting by Dr Samer Mohamed (CCHE, Egypt), Dr Jane Kaijage (Tumaini la Maisha, Tanzania) and Dr George (College of Medicine, Blantyre, Malawi). For example, clinicians in Tanzania use home-made smoothies as supplements during cancer care, whereas Malawi relied upon supplements provided by the acute malnourished ward. Education of staff has been reported as a barrier to nutritional intervention [9]. The International Initiative for Paediatrics and Nutrition (IIPAN) has established an intensive programme in Africa to begin to close this gap in clinical care. Happiness Ndifon, a nutritionist from Cameroon Baptist Convention Health Services, Cameroon, presented how she had implemented a nutrition programme in Cameroon after attending a 2-week intensive training course at an IIPAN training site (South Africa).

Finally, the oncology team from 57357 Children’s Hospital in Egypt presented on the centre’s research. Topics included the role of nutritional therapy and sensitisation to radiotherapy (Dr Ahmed El-Saka), high aflatoxins in Egyptian food (Dr Afaf Amin) and the important role of breastfeeding as part of immunomodulatory therapy (Gihan Fouad).

In conclusion, the workshop established that there is a need for collaborative, prospective studies on nutritional status in PCU in Africa and, by including MUAC, standardised assessment can be achieved. Education of staff members and synergy among nutritional groups within hospitals, particularly with existing malnutrition clinics, is a pressing need for PCU in Africa. Moreover, PCU need financial and product support to be able to increase nutritional interventions. The request for similar workshops to improve nutritional care in their PCU in future years was received, with the first workshop planned in Kenya and subsequent plans for the next SIOP Africa congress to be held in Kampala, Uganda, in 2021.

## Progress in optimising management of the most curable childhood solid tumours

### Burkitt’s lymphoma

Catherine Patte (Institut Gustav Roussy, France) reported the latest results of the international intergroup randomised trial, the ‘Inter-B-NHL Ritux 2010 trial,’ run in eight European countries, Australia, Canada, Hong Kong and the USA. This showed that the addition of rituximab to a standard backbone of intensive chemotherapy (the Lymphomes Malins B (LMB) regimen) improved event-free survival (EFS) from 84% to 92% for advanced stage B-cell lymphoma and B-cell acute leukaemia, and it is now used as a standard in high-income countries (HICs) [11]. Although the longer term immune status of these patients is still under evaluation, a few long-lasting profound B immunodeficiencies have been observed. Hence, rituximab is not currently recommended in addition to chemotherapy in patients with low (stages I and II) or intermediate (stage III with low lactate dehydrogenase level) stages who have an EFS > 97% with no expected late sequelae related to chemotherapy. In particular, the benefit of rituximab in sub-Saharan countries, where most children are malnourished and more susceptible to infections, must be evaluated before recommending its use. C Patte also reported results of GFAOP studies showing that LMB-based chemotherapy is feasible in sub-Saharan countries and that initial dose intensity is crucial. H Abdel Rahman (National Cancer Institute, Cairo University and CCHE, Egypt) showed in a prospective study of fluorodeoxyglucose positron emission tomography (FDG-PET) for assessment of residual masses in mature B cell non-Hodgkin lymphoma that it is not specific enough and recommends the continued need for histological confirmation to avoid unnecessary treatment escalation. Dr Jenny Geel (University of Witswatersrand, Johannesburg) described efforts to improve overall survival for childhood cancer in South Africa, a country with 16.5 million children aged <15 years. They are taking a disease-by-disease approach to implement a unified national diagnostic and treatment protocol, aiming to improve survival rates, decrease toxicity, and understand and control the costs. The first tumour chosen is Hodgkin’s lymphoma. E Moussa (National Cancer Institute, Cairo University and CCHE, Egypt) developed the controversies in the treatment of Hodgkin Lymphoma. Posters reported on North African single centre result in NHL and high-dose (HD), focussing on unusual sites and causes of treatment failures (toxic deaths and malnutrition). One poster on Burkitt highlighted the benefit of a second pre-phase before starting the induction chemotherapy. Another one confirmed the value of PET after two courses of chemotherapy as a predictor of outcome in HD. Prof Peter Hesseling (Stellenbosch University, South Africa) presented results indicating a risk of decreased fertility in girls receiving important doses of cyclophosphamide for the treatment of Burkitt lymphoma.

### Wilms tumour

In the session on renal tumours, Prof Kathy Pritchard-Jones (University College London, UK) gave an update on optimisation of clinical risk stratification for the treatment of Wilms tumour (WT) in the SIOP Renal Tumours Study Group new ‘UMBRELLA’ protocol following further analyses of the previous randomised trial that had recommended omission of doxorubicin from postoperative chemotherapy for all stage II/III intermediate-risk histology WTs [12]. Pending the outcome of ongoing molecular biomarker research, focused on the somatic gain of chromosome 1q, she showed evidence for excess relapse in tumours with volume greater than 500 mL after pre-operative chemotherapy, when the histological subtype was mixed or regressive subtype. It is now recommended that these tumours continue to be treated with doxorubicin included in postoperative chemotherapy [13]. Modest doses of doxorubicin are also now recommended for children with micrometastases visible only on computed tomography (CT). However, it is still acceptable to do staging using a chest X-ray, which is widely available in LMICs.

**Figure 2.**
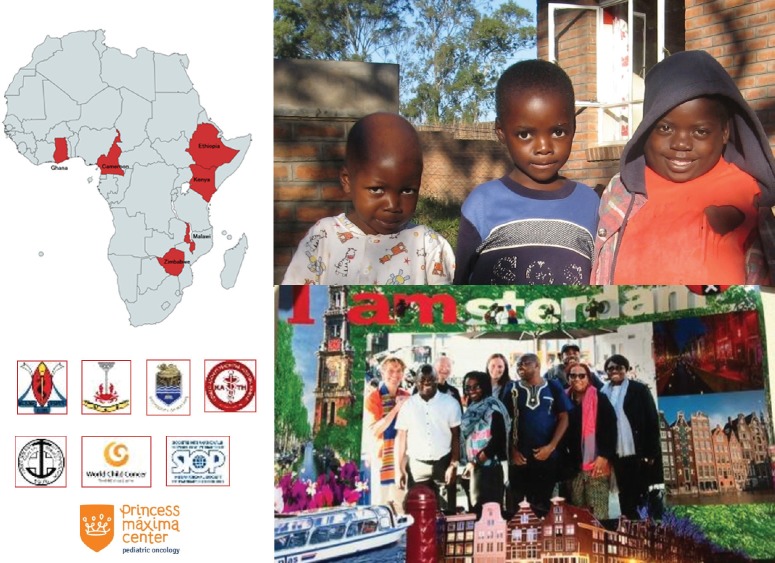
The Collaborative Wilms Tumour Africa Project brings together healthcare providers, hospitals, academic institutions, professional societies, and non-governmental organisations to improve cancer care and outcomes in several countries of Africa.

The collaborative WT Africa Project, presented by Dr Francine Kouya (Cameroon Baptist Convention Health Services, Cameroon) has implemented an adapted WT treatment guideline in sub-Saharan Africa, based on SIOP Renal Tumours Study Group (RTSG) protocols, as a multi-centre prospective clinical trial. Seven centres in Malawi, Cameroon, Ghana and Zimbabwe are participating (Figure 2). The collaborative project’s primary aims are to improve survival to more than 50% by reducing abandonment of treatment and death during treatment to below 10%. A retrospective, baseline evaluation of end of treatment outcome was done for a 2-year period prior to the introduction of the guideline. Compared to the baseline evaluation, abandonment of treatment decreased from 23% to 13% (*p* = 0.03) and death during treatment decreased from 21% to 13% (N.S.). End-of-treatment survival without evidence of the disease increased in the first 2 years of the project from 52% to 68% (*p* = 0.01) [14].

This collaboration, using relatively simple and low-cost interventions has strengthened the local healthcare teams’ knowledge and use of sustainable tools to decrease abandonment of treatment and reduce toxic deaths. The increase in survival without evidence of disease at the end of treatment is expected to translate into improved long-term survival. The group is currently analysing the data of the first 4 years of the project and preparing to start phase II of the project in January 2020. This is expected to include some modifications to postoperative chemotherapy and a uniform relapse strategy. The group is also developing supportive care for children with cancer in Africa (SUCCOUR), a project to improve supportive care for children in sub-Saharan Africa. Centres in Africa wishing to join these projects are most welcome.

### Supportive care for children with cancer in Africa

Improved supportive care has the potential to benefit children with all types of cancer and those in general paediatric care. SUCCOUR is a comprehensive, inclusive project led by doctors and nurses to promote improvements in supportive care. It builds on the lessons learnt from the Collaborative WT Africa Network with step-by-step development and implementation of simple, effective and cost-effective supportive care interventions, giving priority to those with the highest expected impact on child survival [15, 16] (Figure 1). Each site first conducts a baseline evaluation of current practises and outcomes in several areas of supportive care such as febrile neutropenia, nutrition, abandonment and the use of traditional medicine. Gaps in care and best practises will be identified and addressed through educational workshops, advocacy, developing local appropriate supportive care guidelines, rigorous outcome evaluation and development of specific interventions based on the collected local evidence. It will reference the well-developed framework for cause-specific interventions to reduce treatment failure for children with cancer in LMICs (Figure 1).

### Prevention and management of toxicity associated with high-dose methotrexate

High-dose methotrexate (HDMTX), defined as a dose higher than 500 mg/m^2^, is used to treat a range of adult and childhood cancers. Although HDMTX is safely administered to most patients, it can cause significant toxicity, including acute kidney injury (AKI). AKI constitutes an oncologic emergency in patients receiving HDMTX but can be successfully prevented and managed even in LMICs. Monitoring of serum creatinine, urine output and serum methotrexate concentration is used to assess renal clearance, with concurrent hydration, urinary alkalinisation and leucovorin rescue, to prevent and mitigate toxicity. Maintenance of alkaline urine pH is especially important because it prevents methotrexate crystallisation in the urine and greatly reduces the rate of methotrexate entry into urothelial cells, thus protecting the kidney by two distinct mechanisms. Where measurement of methotrexate levels is not available or not available within a clinically useful timeframe, successful management of patients requiring HDMTX therapy depends on using somewhat lower doses (2–3 g/m^2^ instead of 5–8 g/m^2^), meticulous measurement of urine output and mucosal erythema, prevention of vomiting, assuring no loss of IV access during the infusion and frequent measurement of creatinine to allow rapid response to any increase. A recent study from Chandigarh, India, used methotrexate 5 g/m^2^ for children with acute lymphoblastic leukaemia (ALL) in a setting where they could not measure methotrexate levels. Using extra hydration, close monitoring and frequent checks of urine pH and creatinine, they delivered 100 courses of HDMTX without worrisome toxicities [16, 17].

## Importance of asparaginase in treating acute lymphoblastic leukaemia

ALL affects 120,000 people each year worldwide, including children and adults. It can be permanently cured more than 80% of the time with treatment regimens that combine glucocorticoids, anthracyclines, vincristine, mercaptopurine and asparaginase [18]. Scott Howard (University of Tennessee, Memphis, USA) discussed approaches to the most effective use of asparaginase, which rely on minimising the likelihood of initial allergic reactions and having access to at least a second formulation of the drug for those who do react. Native *Esherichia coli* asparaginase (Elspar, Leunase, Kidrolase and others) is on the WHO list of essential medications, while other formulations, currently PEGylated-*E. coli* asparaginase (Oncaspar), and Erwinia asparaginase (Erwinaze), as second-line asparaginase for patients who develop hypersensitivity to *E. coli* asparaginases, are not.

In recent times, there have been problems felt around the world regarding the availability and affordability of asparaginase and question marks have been raised about the quality of some suppliers [1]. In HICs, PEG-*E. coli* asparaginase is used as frontline therapy because it is long-acting and has low rates of hypersensitivity (10%–15%) and silent neutralising antibody formation (1%) than native *E. coli* asparaginase. Most LMICs use the much cheaper native *E. coli* asparaginase, to which allergic reactions (20%–42% of patients with ALL) and neutralising antibody formation (in another 30%–40%) are much common. This means that two-thirds of patients do not attain the required asparaginase depletion unless they have access to a second asparaginase product, usually Erwinia asparaginase. Unfortunately, the supply of Erwinia asparaginase has been limited to HIC, and recent shortages have affected patients even in HIC. When no second product is available, the inability to complete asparaginase treatment increases the risk of relapse. Therefore, minimisation of allergic reactions to the initial form of asparaginase improves outcomes and reduces costs.

The recently published UKALL 2003 trial used PEG-*E. coli* asparaginase in a schedule that included several days of glucocorticoids prior to each dose of PEG-*E. coli* asparaginase in the low-risk and intermediate-risk patients, who had a 1% rate of allergic reaction and excellent event-free survival [19]. Patients on the high-risk arm received several doses of PEG-*E. coli* asparaginase without preceding glucocorticoids and had a reaction rate of 6%, such that, in the whole study, the reaction rate was 2% [19]. This has led to an immediate change in practise, and modification of existing protocols to include glucocorticoids a couple of days before each PEG-*E. coli* asparaginase dose, in the hope of reducing allergic reactions to 1%, thus allowing patients to complete all asparaginase and reducing the need for second-line asparaginase (e.g. Erwinia). Prof Howard discussed five strategies to the choice of first-, second- and third-line asparaginase, and concluded that the most clinically effective and cost-effective strategy is upfront use of PEG-*E. coli* asparaginase with second-line *Erwinia* asparaginase, when available, in the 10%–15% of patients who develop hypersensitivity. The average patient who receives PEG-asparaginase 1000–2500 U/m^2^ has adequate asparaginase activity for 14–24 days, a duration that would require repeated dosing of native *E. coli* asparaginase 2–3 times per week during this interval, or a total of 6–9 doses, to achieve comparable asparaginase activity [20].

### Information systems

All strategies to reduce treatment failure for children with cancer depend on a robust information system to facilitate continuous, relentless quality improvement. The advanced health informatics system of CCHE, hospital 57357, is not affordable in most African settings. Alternative systems adapted to the practical challenges faced in Africa were presented. Prof Scott Howard described Resonance Oncology (www.ResonanceOncology.org), an academically led, cloud-based, cancer information system, available at no cost to centres in LMICs. Baseline risk assessment for abandonment can be stored in the oncology adapted resonance patient centre (RPC) abandonment module and the risk score calculated there. RPC can also contain all the patient’s clinical information, chemotherapy roadmap and appointments, and serves as a unified source of information about the patient’s care and outcomes. The system supports multiple languages, and the ability to produce analytics and visualisations in real time allows sites to quickly and frequently assess the causes of treatment failure by region, country, cancer centre, year of diagnosis or cancer type [21]. When cancer registry data, abandonment risk factors, treatment appointment adherence and outcomes (causes of treatment failure) are collected in real time for all patients, deployment of interventions can be based on local needs and priorities (Figure 1). A prize-winning oral presentation by Jeremie Hassan (Tumaini la Maisha, Tanzania) ‘Increasing Safety and Consistency of Chemotherapy Treatment in Resource-Limited Countries via Excel-Based Prescription Automation’ described the work in Microsoft Excel to create printable chemotherapy prescription smart sheets for common childhood cancer. Eight protocols have been fully automated until now. There were highly interactive discussions with the audience who found it as a very good method to reduce treatment errors that could lead to a greatly improved treatment safely and efficiency.

The surgical session was well attended by different generations of paediatric surgeons and other medical specialities and emphasised the importance of formal multidisciplinary discussion with oncologists and radiotherapists to optimise individual patient care. A major recommendation was to promote the importance of involvement in multicentric studies and surveys. It was proposed that a future conference should be organised with a larger faculty from IPSO, the SIOP surgeons.

## Treatment of brain tumours in childhood

The neuro-oncology session provided the opportunity to address the challenges associated with the development of paediatric neuro-oncology programmes in countries with limited resources. A number of factors affect these efforts, such as lack of awareness of paediatric brain tumours, late diagnoses, limited imaging facilities, absence of paediatric neurosurgical training, lack of expertise in neuropathology, difficulties to access radiation services and absence of multidisciplinary approach. Several solutions have been investigated, and, so far, the most successful experiences are with the development of twinning programmes between institutions in high-income and low-income countries. The use of teleconferences allows face-to-face interactions, and regular reviews and discussions of challenging cases have a major impact on clinical practise.

In this context, Dr Giorgio Perilongo (University of Padova, Italy) discussed the management of paediatric low-grade gliomas, reminding the audience that this condition has been listed in the six diseases targeted by the WHO Global Initiative for Childhood Cancer. Major advances in the understanding of the molecular biology of this condition have happened during the last decade, leading to the development of new strategies targeting the RAS/MAP-Kinase pathway. However, these progresses are unlikely to benefit African patients in the near future, and the management of African patients should take into account a number of factors, including distance from the hospital, side effects of chemotherapy and risk of abandonment. In this context, radiation may have still an important role, in particular, when conformal radiation is available. The management of medulloblastoma is far more complex, as it requires a timely and multidisciplinary approach. Dr Kieran (Boston Children’s Hospital, USA) reviewed the recent progress in the management of this condition and addressed the main factors of success which include access to a paediatric neurosurgery facility, timely referral to the radiation oncology unit, and adjuvant chemotherapy and follow-up provided by an experienced neuro-oncology team. Dr Bouffet provided an overview of paediatric cancers associated with mismatch repair deficiency (MMRD), an often under-recognised condition closely associated with parental consanguinity. MMRD is not exceptional in Africa where consanguinity is common. Children with MMRD develop malignant brain tumours, lymphoma and colon cancers. There is emerging evidence that some MMRD-related solid tumours can be successfully treated with immune checkpoint inhibitors, and the management of cancers associated with this condition may require a specific approach. Dr Zaghloul reported on the ongoing trial of radiotherapy for patients with diffuse intrinsic pontine glioma (DIPG), which suggests that hypofractionated radiation is given over 13 or 15 sessions at a dose of 3 Gy per session (39 or 45 Gy). Both are equivalent (non-inferior) to standard fractionation (54 Gy in 30 fractions). This experience has certainly important implications, in particular, when access to radiation facilities is limited.

## Radiotherapy

In the Radiation Oncology session chaired by Jeannette Parkes (University of Cape Town, South Africa) and Mohamed Zaghloul (National Cancer Institute, Cairo University and CCHE, Egypt) eight important topics were discussed exploring the problems, limitation and future of radiation oncology in Africa. Parkes (South Africa) presented the important issue of the interdependence of radiotherapy and neuroimaging, with the need for services to keep abreast of advances in imaging to improve the quality and accuracy of radiotherapy. Zaghloul (Egypt) presented the situation of radiation oncology in Egypt and on the continent of Africa. The causes of the deficiencies in radiotherapy service availability were widely discussed together with the suggested ideas to improve its level [22]. Egypt, as an example, could showcase the importance of collaboration between governmental institutions, universities, NGOs, international bodies and societies like IAEA, ASTRO, ASCO, ESTRO, PROS (Paediatric Radiation Oncology Society) to improve both the quantity and quantity of radiotherapy to serve African patients [23]. Dorra Aissoui (Habib Bourguiba University Hospital, Tunisia) presented a profile of the positive changes for paediatric radiation oncology that had occurred in her centre during 2010–16. The improvements achieved are expected to reflect upon survival and quality of life for the children treated.

Several presenters from Egypt described clinical and technical advances in treating patients at their centres. Soha Ahmed (Aswan University and CCHE, Egypt) presented the experience at CCHE to salvage children with recurrent ependymoma after re-excision of the recurrence (or without surgery) through reirradiation. She summarised the international as well as CCHE experience and concluded that it is not only feasible but also beneficial in terms of overall survival and progression-free survival. Engy Salah (CCHE) presented the experience of re-irradiation in DIPG patients in their first progression. Comparison of 27 re-irradiated patients with a retrospective matched cohort of 27 patients receiving best supportive care demonstrated safety and suggested efficacy. Further presentations described a new technique, deep inspiratory breath hold (DIBH) in mediastinal Hodgkin’s Lymphoma in adolescents. Haytham Shaheen (CCHE) presented a full description of the technique, its scientific background and advantage using the novel surface scan (Catalyst) together with cone beam CT. Shaheen convinced the audience of the simplicity, accuracy and efficiency of the system. Hany Ammar (CCHE) compared the different radiotherapy techniques during treatment with DIBH. The DIBH offer much superior dosimetric distribution than free breathing.

Volumetric modulated arc therapy was shown to be more accurate and to provide improved tumour coverage with reduced dose to surrounding normal structures, while requiring reasonable monitor units and time on the machine compared to intensity-modulated radiotherapy and conformal radiotherapy. Finally, Caroline Elmaraghy (CCHE, Egypt) presented the CCHE experience in treating focal brainstem glioma. In a retrospective study, 72 patients were treated either by careful watching, chemotherapy or radiotherapy according to certain criteria depending on symptoms, site, size and progression of the tumour. Although those who received radiotherapy had slightly better overall survival and progression-free survival, the differences were not statistically significant. The interaction between the audience and the speakers were high with exchanging ideas and experiences.

Professor El Beltagy, professor of neurosurgery, Cairo University and Head of Neurosurgery, CCHE, Egypt presented the rationale for and the official launch of the African paediatric neuro-oncology society (APNOS). Many problems are encountered in the diagnosis and treatment of childhood brain tumours in Africa due to lack of resources and scarcity of appropriately trained neurosurgeons and other physicians. There is a deficiency in paediatric neurosurgeons in Africa with a median number of neurosurgeons per 100,000 population of 0.01. Not only is there a severe shortage of trained neurosurgeons, but also equipment, funding and teaching programmes. These signify poorly developed health systems and uneven distribution of neurosurgical and radiotherapy facilities in many countries and across the continent. The consequences are the high mortality and morbidity rates seen today from conditions requiring neurosurgical interventions, with a delay in diagnosis and complicated clinical presentations.

After successful activities over the past 3 years, including workshops for neurosurgery and training programmes for African doctors, the decision was taken to initiate the APNOS and announce its creation during the SIOP Africa 2019 conference. APNOS is an initiative to strengthen collaboration between African countries to improve diagnosis and management of paediatric brain tumours between experts in neuro-oncology and neurosurgeons from many African countries. APNOS has 63 members and board members from different African countries (Egypt, Morocco, Algeria, Tunisia, Libya, Sudan, Nigeria, Zimbabwe and Kenya). APNOS aims to be a leading model of collaboration towards a childhood brain cancer-free Africa, through establishing, facilitating and supporting the paediatric neuro-oncology services across Africa through continuous training, education and capacity building to help alleviate the suffering of African children with brain tumours. The first APNOS congress is planned for the first half of 2020, to be held in Cairo, Egypt, as the first of a series of planned semi-annual neurosurgery workshops in Egypt, Morocco and Sudan, covering different topics including hydrocephalus, endoscopic surgery and tumour surgeries. APNOS is also supervising the neurosurgical fellowship programme (CCHE-57357; starting 2019). In the field of radiation oncology, APNOS collaborated with the Paediatric Radiation Oncology Society (PROS) to help the implementation of the first practical radiotherapy course in 2016 (CCHE-57357), and continues to collaborate on the medical biophysics training programme and preparation for the paediatric medical biophysics 57357 fellowship together with the radiation oncology fellowship programme (CCHE-57357; starting 2019). There is also an ongoing Paediatric Oncology fellowship programme for neuro-oncology training (currently ongoing at CCHE-57357). It is a 30-month fellowship programme in collaboration with the Dana Farber Cancer Institute, including a 6-month paediatric neuro-oncology subspecialty training. Through this programme, there are two African graduates so far from Ethiopia and Kenya.

## Report of the joint session with parents

CCI Africa was established a year ago in Johannesburg, under the supervision of Ruth Hoffman, the CCI global president. The SIOP Africa congress was the first meeting held in partnership with CCI Africa, with an integrated ‘parent track.’ The programme was led by Ruth Hoffman and the board of CCI Africa, which has seven members from South Africa, Zimbabwe, Uganda, Kenya, Nigeria, Ghana and Egypt, with Carl Queiros as elected President of the CCI African Regional Committee. There were presentations from representatives of parent groups from South Africa, Zimbabwe, Nigeria, Kenya and the Alexandria group of childhood cancer care (AGCCC) in Egypt. Three members of AGCCC presented on the Egyptian experience of founding the first support group for children with cancer and their families in Alexandria city, describing how they mobilised all the potential powers of the community as well as NGOs to establish the Hospitality House Caring for Cancer Children. There was a very fruitful discussion and dialogue between two survivors: one from Kenya (Mr Sydney) and one from Cairo, Egypt (Mr Mahmoud). Overall, this first joint session between CCI Africa and healthcare professionals involved in SIOP Africa was very fruitful and pointed the way for other regions to achieve better care and support for cancer children and their families in the African setting.

## Conclusion

This SIOP Africa congress highlighted many positive actions in improving care and survival rates for children with cancer in Africa. It provided an important forum for policy discussions with WHO in relation to their global mapping initiative of childhood cancer services, that commenced with African countries. All but six African countries responded, but some stated they have no specific services for cancer in children and young people. WHO’s 2015 ambition was to reduce deaths from four non-communicable diseases by 25%. Cancer was not mentioned specifically, although it was included in the overall target. Now the WHO 2018 Global Initiative for Childhood Cancer has a specific target to improve childhood cancer survival rates in all countries to at least 60% by 2030. This target is tractable by the knowledge we have now.

The conference showcased many twinning initiatives that will contribute to sustainable improvements, such as the Francophone GFAOP that has helped to establish 20 childhood cancer units in 16 countries, offers a 1-year diploma course from the University of Paris Sud and has trained 240 doctors and nurses who have treated >8,000 children. The business meeting of SIOP Africa highlighted that governments need to listen to the issues and the potential solutions provided—only 18 African countries have cancer plans identified through the survey and only six mentioned the specific needs of children with cancer. Furthermore, the importance of partnership working with parents’ organisations cannot be ignored—while CCI Africa is now a visible improvement partner, there are large parts of Africa without parents’ organisation registered with CCI. The SIOP Africa 2019 conference has provided model solutions that now need to be adopted at scale. We hope that our governments are listening!

As our closing remarks, we would like to dedicate this conference report to three wonderful healthcare professionals and human beings, who lost their lives on their way home from the conference, full of new ideas and pride in what they had achieved so far. We hope that all those who read this report will be inspired by their work and will continue their excellent work to improve cancer care for children in Africa. https://siop-online.org/a-tribute-to-jayne-bella-grace/

## Conflicts of interest

All authors declare that there are no conflicts of interest in relation to this article.

## Funding

This article received no specific funding.

## References

[1] Lam CG, Howard SC, Bouffet E (2019). Science and health for all children with cancer. Science.

[2] SIOP Africa conference website. http://siop-africa2019.com/.

[3] Childhood cancer international–Africa website. https://www.childhoodcancerinternational.org/cci-global-network/africa/.

[4] Day S, Hollis R, Challinor J (2014). Baseline standards for paediatric oncology nursing care in low to middle income countries: position statement of the SIOP PODC nursing working group. Lancet Oncol.

[5] Morrissey L, Lurvey M, Sullivan C (2019). Disparities in the delivery of pediatric oncology nursing care by country income classification: international survey results. Pediatr Blood Cancer.

[6] SIOP basic nursing standards. https://siop-online.org/baseline-nursing-standards/.

[7] Eskander HG, Morsy WYM, Elfeky HAA (2013). Intensive care nurses’ knowledge & practices regarding infection control standard precautions at a selected Egyptian cancer hospital. J Educ Pract.

[8] Schoeman J (2015). Nutritional assessment and intervention in a pediatric oncology unit. Indian J Cancer.

[9] Schoeman J, Ladas E, Rogers P (2018). Unmet needs in nutritional care in African paediatric oncology units. J Trop Pediatr.

[10] Ladas EJ, Arora B, Howard SC (2016). A framework for adapted nutritional therapy for children with cancer in low- and middle-income countries: a report from the SIOP PODC nutrition working group. Pediatr Blood Cancer.

[11] Minard-Colin V, Auperin A, Pillon M (2016). Results of the randomized Intergroup trial Inter-B-NHL Ritux 2010 for children and adolescents with high-risk B-cell non-Hodgkin lymphoma (B-NHL) and mature acute leukemia (B-AL): Evaluation of rituximab (R) efficacy in addition to standard LMB chemotherapy (CT) regimen. ASCO.

[12] Pritchard-Jones K, Bergeron C, de Camargo B, SIOP Renal Tumours Study Group (2015). Doxorubicin omission from the treatment of stage II–III, intermediate-risk histology Wilms’ tumour: results of the SIOP WT 2001 randomised trial. The Lancet.

[13] van den Heuvel-Eibrink MM, Hol JA, Pritchard-Jones K (2017). International society of paediatric oncology—renal tumour study group (SIOP–RTSG). Position paper: rationale for the treatment of Wilms tumour in the UMBRELLA SIOP-RTSG 2016 protocol. Nat Rev Urol.

[14] Israels T, Paintsil V, Nyirenda D (2018). Improved outcome at end of treatment in the collaborative Wilms tumour Africa project. Pediatr Blood Cancer.

[15] Israels T, Molyneux E, SIOP Africa–PODC Collaborative Wilms Tumour Project Group (2019). Lessons learned from a multicentre clinical trial in Africa. Nat Rev Clin Oncol.

[16] Davidson A, Howard SC (2018). Delivering modern anticancer therapies in low- and middle-income settings: we can be evidence based. Pediatr Blood Cancer.

[17] Vaishnavi K, Bansal D, Trehan A (2018). Improving the safety of high-dose methotrexate for children with hematologic cancers in settings without access to MTX levels using extended hydration and additional leucovorin. Pediatr Blood Cancer.

[18] Pui CH, Yang JJ, Hunger SP (2015). Childhood acute lymphoblastic leukemia: progress through collaboration. J Clin.

[19] Vora A, Goulden N, Wade R (2013). Treatment reduction for children and young adults with low-risk acute lymphoblastic leukaemia defined by minimal residual disease (UKALL 2003): a randomised controlled trial. Lancet Oncol.

[20] Vrooman LM, Stevenson KE, Supko JG (2013). Postinduction dexamethasone and individualized dosing of Escherichia Coli L-asparaginase each improve outcome of children and adolescents with newly diagnosed acute lymphoblastic leukemia: results from a randomized study—Dana—Farber cancer institute ALL consortium protocol 00-01. J Clin Oncol.

[21] Howard SC, Zaidi A, Cao X (2018). The my child matters programme: effect of public-private partnerships on paediatric cancer care in low-income and middle-income countries. Lancet Oncol.

[22] Bishr MK, Zaghloul MS (2018). Radiotherapy availability in Africa and Latin America: two models of low/middle income countries. Int J Radiat Oncol Biol Phys.

[23] Zaghloul MS, Bishr MK (2018). Radiation oncology in Egypt: a model for Africa. Int J Radiat Oncol Biol Phys.

